# Citizen perspectives on the use of publicly reported primary care performance information: Results from citizen‐patient dialogues in three Canadian provinces

**DOI:** 10.1111/hex.12902

**Published:** 2019-05-10

**Authors:** Sharon Johnston, Julia Abelson, Sabrina T. Wong, Julia Langton, Mathew Hogel, Fred Burge, William Hogg

**Affiliations:** ^1^ Department of Family Medicine University of Ottawa Ottawa Ontario Canada; ^2^ CT Lamont Primary Health Care Research Centre Élisabeth Bruyère Research Institute Ottawa Ontario Canada; ^3^ Department of Clinical Epidemiology & Biostatistics, Centre for Health Economics and Policy Analysis McMaster University Hamilton Ontario Canada; ^4^ Centre for Health Services and Policy Research University of British Columbia Vancouver British Columbia Canada; ^5^ School of Nursing University of British Columbia Vancouver British Columbia Canada; ^6^ Department of Family Medicine Dalhousie University Halifax Nova Scotia Canada

**Keywords:** accountability, performance reporting, primary care, public participation

## Abstract

**Objective:**

Performance measurement and reporting is proliferating in all sectors of the healthcare system, including primary care, despite a dearth of evidence on how the public uses reports on primary care performance. We explored how the public might use this information, to guide the development of effective reporting systems for primary care.

**Methods:**

We conducted six full‐day deliberative dialogue sessions with a purposive sample of 56 citizen‐patients across three Canadian provinces (British Columbia, Ontario and Nova Scotia). Participants identified how they would use publicly reported performance data. We conducted a thematic analysis of the data by region.

**Results:**

Common uses for primary care performance information emerged across all sessions. Participants most often discussed the utility of this information for community advocacy and participation in health system decision making. Similar barriers for using performance information to choose a primary care provider were identified in each region including the perceived lack of choice of providers and the high value placed on relationships with current providers. Finally, the value of public performance reporting in enhancing trust that people would receive good care was also a common theme.

**Conclusions:**

Citizen‐patient perspectives highlight that public reporting on primary care performance could promote the health system's responsiveness by enabling public engagement in decision making at the community level. The role of public reporting in promoting trust rather than empowering patient choice may reflect unique elements of the Canadian health system's context.

## 1 INTRODUCTION

The increasing focus on patient experience, accountability, cost‐effectiveness and quality improvement in healthcare is driving investment into healthcare system performance measurement and reporting.^1^, ^2^, ^3^, ^4^, ^5^, ^6^ Public reporting has many intended purposes, including quality improvement,^1^, ^7^ public accountability,^2^, ^8^, ^9^, ^10^, ^11^ patient engagement^12^, ^13^, ^14^ and informed decision making.^15^ However, limited and mixed evidence exists on how public reporting of performance information influences the behaviours of various stakeholders.^1^, ^14^, ^16^, ^17^ This is especially true in primary care where there is less evidence on public reporting of performance compared to hospital and specialist sectors.^18^, ^19^, ^20^, ^21^ Understanding how the public uses primary care performance information may be helpful in shaping effective reporting strategies.^22^, ^23^ Countries with stronger primary care systems have healthier populations, as well as a more equitable distribution of health across the population.^24^ As primary care is becoming increasingly complex,^25^ helping the public understand how their primary care system performs is an important part of understanding their country's overall healthcare system.^26^

Countries like the UK and Australia have conducted nationwide public reporting on primary care performance for more than a decade. They are now sharing early lessons learned on the optimal format and ways of presenting data to the public, including emphasizing easily accessible data for initially engaging the public.^27^, ^28^, ^29^ Smaller scale studies of the utility of well‐designed reports in the USA have shown that people choosing healthcare providers can engage with quality of care information which is easy to understand to select high‐value choices.^30^ Unique features of Canada's primary care systems, such as its public funding, expectation that healthcare is a government responsibility, and shortage of primary care providers in some communities,^9^, ^31^, ^32^ suggest the value and type of publicly reported primary care performance information could be different in this context. As a greater number of provinces and health regions are publishing measures of primary care performance, there is a need to explore how people might use publicly reported primary care performance information to inform the design of these emerging reporting initiatives. In this study, we conducted a series of citizen‐patient deliberations in three Canadian provinces focused on the uses of and optimal methods for primary care performance measurement and reporting.

## 2 METHODS

This project was embedded within a larger programme of research comparing primary care performance in three distinct regions in the provinces of British Columbia, Ontario and Nova Scotia. The regions were selected for their varied approach to primary care reform and performance measurement and reporting over the past decade (unpublished data)^33^ and because they were identified in 2013, the first year of the study, as health regions with similar socio‐demographic profiles.^34^ The regions also had marked differences in ratio of general practitioners or family physicians to the general population, rates of hospitalization for ambulatory care sensitive conditions, often used as a marker of primary care performance in managing certain chronic conditions like diabetes and asthma, as well as a large spread in proportion of the population with a regular primary care provider compared with the Canadian provincial averages.^35^ See Table 1 for characteristics (eg, population, health status) of the three study regions.

**Table 1:** Key characteristics of the three study regions

	Fraser East, BC	Eastern Ontario Health Unit, ON	Central Zone, NS
Estimates of population,^53^ n	300 724	205 982	447 050
General/Family physicians,^54^ rate/100 000	99.4	91.9	150.6
Specialist physicians,^54^ rate/100 000	56.8	34.7	189.5
% has a regular healthcare provider^55^	84.1	90.5	87
Perceived health^55^			
% very good or excellent	59.3	60.8	63
% fair or poor	11.5	15.3	10.6
% obese^55^	32.2	29	29.6
% with arthritis (15 y and over)^55^	23	22.8	22
% with diabetes^55^	7.2	9.5	8
% with high blood pressure^55^	13.4	21.8	16
% with moderate or severe pain or discomfort^56^	13.5^E^	15	11.6
Ambulatory care sensitive conditions hospitalization rate (2011 standard population), age‐standardized rate/100 000^54^	383	506	249

Six day‐long deliberative dialogue sessions were held between January and May 2016, two in each of three regions. These sessions engaged people living in these different communities to explore whether common uses for primary care performance information would emerge across the regions.

Deliberative participatory methods, a well‐established approach for engaging members of the public in complex topics, were chosen because they use structured facilitation to present balanced background information and enhance participants' understanding of the topic. Adequate time is given to foster learning, reflection and reasoned engagement.^36^, ^37^, ^38^ We sought participants who had different levels of experience with the healthcare system in case experience impacted how people might use publicly reported performance information. Accordingly, one deliberation event in each region was for people with multiple medical conditions (Session 1) and the second event was for those with two or fewer medical conditions (Session 2). The study was approved by the institutional review boards of the University of British Columbia, the Nova Scotia Health Authority, the Ottawa Hospital, and Bruyère Continuing Care in Ottawa.

### 2.1 Recruitment

Individuals aged 18 and older who participated in a waiting room patient experience survey at their primary care practice were also asked for their consent to be contacted for related research opportunities. Participants were primarily recruited from a convenience sample of these consenting survey participants, and we obtained their age and medical conditions from the waiting room survey data. We had to recruit additional participants in Ontario (1) and Nova Scotia (9) through a study recruitment advertisement posted on a regional job and volunteer opportunity website to augment the number of people with no or one medical condition. We sought diversity in age, gender, types of chronic conditions, and the practice with which participants were affiliated. Participants had to speak English. They were offered a $75 honorarium, meals during the event, and reimbursement for transportation costs.

### 2.2 Structure of the deliberation

Each 1‐day deliberation was held in a central location within the study regions and facilitated by an experienced moderator. Prior to each session, participants received an information package detailing the project objectives and background information about primary care performance measurement, including the rationale for public reporting and the specific study questions for discussion, definitions of key terms such as the primary healthcare system, and examples of most commonly used performance domains in primary care, such as access, along with examples of different indicators which could be used to evaluate performance in that domain. The agenda for the session was structured around three discrete topics: (a) prioritizing primary care performance dimensions and indicators for public reporting (See Appendix S1); (b) uses of performance information; and (c) effective reporting formats. We presented scenarios, illustrative examples, and reviews of reporting in Australia, the UK, Canada and international comparisons throughout each session.

The second topic is the subject of this analysis and was informed by a rich discussion of the type of information which could be publicly reported to enable participants to contemplate uses for information they might not have considered before the dialogue sessions. The opening hour was spent reviewing the background material and orienting participants to the concepts of primary healthcare and performance measurement using interactive case discussions to both familiarize the participants with the concepts and help them identify and share their own experiences with and opinions on evaluating healthcare performance. Content experts in primary care performance measurement, patient experience, public performance reporting and deliberative dialogue methodology were present and involved in facilitating the discussion to ensure an interactive group discussion with opportunities for each participant to share their experience and learn.

### 2.3 Analysis

Each deliberation session was recorded and transcribed. Observer notes were taken, and a team debrief was held after each session. The transcripts were read in their entirety using crystallization and immersion methodology^39^ by two team members experienced in qualitative data analysis (SJ, MH), one of whom attended each session and carried out data cleaning for any transcription errors (SJ). A coding template was inductively developed by the interdisciplinary analysis team (SJ, MH, JA, SW, JL), broadly informed by the study objectives and the initially emerging concepts from the in‐depth transcript review. Two team members (SJ and MH) independently coded each of the transcripts. The two then compared coding of the entire transcripts to ensure agreement on all coded segments. Coded segments were then entered into NVivo qualitative data analysis software (v.10).^40^ Coded segments were reviewed by the analysis team to identify shared themes (arising across all or multiple groups, supportive statements from separate participants), as well as conflicting statements, and unexpected information. Shared themes were also mapped to identify if any were specific to either the medically complex group of participants or the less medically complex groups. The team had extensive expertise in different substantive (performance measurement, patient experience, primary care), methodological (qualitative and deliberative methods) and clinical (family medicine, nursing) research areas. We carried out a thematic analysis across all deliberative dialogues as well as assessed for similarities and differences by region and for the two groups of patients (less complex and medically complex).

## 3 RESULTS

We involved 56 participants with a range of ages from 20 to 81 years old and no medical conditions to ten different conditions. (See Tables 2 and 3). We had a balance of men and women across the groups of each region except in Nova Scotia where all but two participants were women. Several shared themes highlighting how individuals might use publicly reported primary care performance information emerged across all three regions and six dialogue groups: to support collective health system decisions, to select providers, and to seek the best care for oneself or one's family. Additionally, some participants also noted a role for publicly reported primary care performance information in quality assurance and promoting trust. That is, while the public may not use or engage with all performance information, it was reassuring to know that performance was being measured and monitored. No recurring themes were limited to or significantly more prominent within either the medically complex or less complex groups.

**Table 2:** Demographic characteristics of participants

	Session 1			Session 2		
	BC	ON	NS	BC	ON	NS
Number of participants	14	10	6	6	11	11
No of female	7	5	5	2	8	10
Age in years						
Mean (SD)	64.1 (11.9)	61.4 (10.9)	56.7 (12.5)	NA^a^	NA^a^	NA^a^
Number of conditions						
Mean (SD)	4.9 (2.1)	5.4 (2.4)	4.7 (2.5)	1.3 (0.5)	0.7 (1.1)	0.3 (0.5)

a Data not available for 13 of the 27 less medically complex participants who were recruited through the online volunteer add. For the 14 participants with age data, the mean was 57 with age range from 20 to 80 y.

**Table 3:** Examples of chronic conditions distribution in medically complex groups

Number of participants with:	BC Session 1 Total participants 14	ON Session 1 Total participants 10	NS Session 1 Total participants 6
Hypertension	8	5	2
Depression	7	3	3
Cardiovascular disease	6	4	1
Osteoarthritis	10	4	5

### 3.1 Collective health system decisions

The most frequent theme was that publicly reported primary care performance information could be used to support collective health system decisions, strategic decisions about health services and policy.

The most common activity participants raised in relation to using performance information in each of the dialogue sessions was advocating for better primary care for their community, where they lived and expected to receive healthcare. As one participant noted, public information could be a precursor to advocacy; “I guess seeing something like this gives people the ability to start conversations when you go home…, which creates a social awareness of issues… Because if I don't know it's an issue, I'm not going to deal with it.” [BC_2] Similarly, another participant from a different region shared that “…the people in our community don't realize that it's not like this everywhere else. … And I think that's something that we need to get out there so people have expectations, and expect more from our healthcare system than we get in this rural community.” [ON_1]

Participants shared that performance information on their region, especially poor performance compared to other regions, would motivate them to hold their elected representatives accountable. As one participant noted, “And if you've got some districts performing better than other districts and the public knows that, that's where the pressure comes from to change…” [BC_1] This theme was repeated across each group, in the words of one participant, if a website reported “that we have to wait eight days to see a doctor… then they can bring that to the government or, you know, whoever's in charge and, like, protest…and so, it may be not be useful to you as a specific individual, but as a whole we can work together …and push in the right direction.” [NS_2]

An important contrasting perspective was expressed by a few participants in each region, who felt that individuals are not empowered to change their communities, and, rather, some communities might be made more vulnerable from public performance reporting.

On one level, I want to know. On the other level, what can I do about it if it's the only clinic, and if I were to put myself in the shoes of the practitioners and see this and say, ῾…they don't like me, I'm going [to] get up and go.’ You know. ῾I'm going to go elsewhere where people do and because they need me.’ So, yeah, I can see it being dangerous, as being a knife with two edges. [ON‐1]

In each region, one or two participants raised the importance of reporting on all communities in the province to make people aware of inequities and promote lobbying on behalf of those regions least well served, particularly rural and northern communities. This advocacy role was described by one participant; “[i]f you head up farther north and your access to healthcare gets worse and worse and worse, and I think you could make that public and accessible for the general public to see [it] becomes more of a real issue for everybody to deal with and to improve those people's quality of life and access to healthcare.” [BC_2] However, this was debated with some participants feeling uninterested in distant regions, or not empowered to influence their services.

Several participants saw public performance information as helpful in holding the government accountable for public spending on healthcare: “As a taxpayer I certainly would want to know where our dollars that I'm paying into this infrastructure—where is it going. …That's of interest to me as a taxpayer, as a citizen.” [NS2] Public performance reports could help them understand where their tax dollars were going and how they were being used.

### 3.2 Selection of providers

The use of performance information to select providers was raised in each region but with many fewer supportive statements than for collective health system decision involvement. Several participants in each region noted a significant role for primary care performance information when moving to a new area.

When the population is moving around, they’re looking, especially the new families … before they make their decision on settling down … they want to make sure they’re going to get the services. Is there a clinic or doctor's office? Is it available? Is it available on weekends? Are the schools available? Are there other places, parks and recreation and things like that? … They’re spending a whole lot of money buying a house or renting a house and they want to make sure that these services are available. [ON_2]

Some participants also noted that better comparative performance information might encourage people to travel outside their immediate region for better care.

If I'm moving to an area and the primary practitioner there is not what I'm looking for, I have the option then of saying, ‘Am I going to deal with someone who doesn't have good quality of care, or am I going to make the difference and go to another town and find a doctor?’ I'm making that choice. I'm in control of it, not the proximity of where I live. [ON_1]

However, participants across all six dialogues identified several barriers to using primary care performance information to choose a provider. The dominant obstacle raised in each group was participants' perception of having minimal choice of providers. “You rate a doctor, you don't like the rating, you have nowhere else to go.” [BC_1] Breaking or changing a relationship with a provider was also seen as requiring a significant effort. “…most people who do have a GP or are connected with their GP already to some extent, it's challenging for them to go to another GP if they did get a bad rating.”[NS_2]

Additionally, in each of the regions there was some negative feedback about promoting a consumer culture or “doctor‐shopping” by publicly comparing the performance of primary care providers. Some people viewed the movement of patients from one provider to a “better performer” as exacerbating inequities. “What kind of frightens me is if you get it from practice to practice or doctor to doctor the thing is people say, ‘Well, I'm not going to go see that doctor anymore. I'm going to go try that one over here.’ So, doesn't that put an overload on another area, and less of a load here...” [NS1] Participants also noted the potential of harming certain communities, such as rural areas.

… one of the things I worry a little bit about especially being part of a rural community where access usually is an issue because we have a very limited number of physicians… when we sort of almost pit one area against another is that what is the risk to the community of losing a physician who cannot perform better because perhaps they’re the only one of two in an area, and can't physically take on more clients. [ON_1]

The use of public performance information to compare and select a better provider was seen as potentially harming high‐performing regions and poor‐performing regions.

### 3.3 Advocating for one's own healthcare

There were diverse perspectives on how people would use publicly reported performance information in making care choices or managing health with their primary care providers. In each region, multiple participants stated they would trust their own positive experience at their practice over published reports of poor performance for their own provider, discounting poor performance ratings for their provider as related to other patient factors, not their provider's actions. “…[T]he best way to get the good feel of how it is, is to experience going to that clinic to know for sure that that is the place, that you may want to go. … because you might go to the clinic, enjoy the service. Somebody else might go and have a bad experience. So, we have to be careful of that.” [ON_2] However, some said they would feel enabled to advocate for themselves if poor performance reports aligned with their personal experience.

A few participants in each region noted that performance information could help them advocate for better care by improving their knowledge of the care they should be receiving (eg, preventive care or cancer screening measures), potentially helping them understand what good care would be for them. “…if I saw that they had low screening rates, if it was publicly provided information, then I could be, like, ‘Oh, I didn't know I was supposed to have that screening. Now I know that I should ask.’ But if that information's not provided, then I don't know that that's something I'm supposed to be screened for.” [NS_2]

### 3.4 Promoting trust

While many participants did not expect to engage with the performance information themselves, they believed their care would benefit from public reporting of it. A few participants suggested that many in the general public would not have the skills to understand performance data and several specified that efforts would be needed to explain the significance of performance results. “What's the difference between 74 percent and 64 percent…when you show the results you will have to educate people about that part too.”[ON1_1] However, they felt it would be reassuring to know that poor quality would be publicly identified and addressed by others. “The information that you gather is only relevant to the things that interest us, but the importance of the information gathered is so important for them to know that they have to be [accountable] …They can't get away with anything if it's reported on and they don't know who's going to be reading it.” [BC_1] A few participants in each region raised that public performance reporting would also advance people's healthcare by being available for their providers to act on. “I think that the nurse practitioners or the primary caregivers would be able to use the information more than we would, then that would benefit us tremendously.” [ON_2] They discussed a desire to trust their clinicians and have others oversee and ensure quality. Some felt the professionals themselves or their regulatory colleges were the appropriate targets for public reporting as they could act to ensure high quality was achieved.

Several participants also stated they would use publicly available information on a provider's continuing professional development to reassure themselves that they would get good care from the clinician.

Being a patient, I don't know how good the physician is up‐to‐date with the processes that are already in place. Has he been re‐educated? Does he follow with new procedures and everything else? The patient does not know this…I want to make sure I get access to a physician that's right up there… I think it gives me more assurance that I'm seeing the proper person or doctor. [ON_2]

Most participants finished the day stating they wanted to know that primary care performance information would be publicly reported.

## 4 DISCUSSION

The perspectives by participants in these three regions contribute to the sparse literature on how public performance reporting in primary care may influence the public's behaviour. Despite regional differences in primary care resources and rural and urban populations, similar potential uses for public performance information were raised in each region, as well as barriers or concerns over its use. These themes were shared by both experienced patients within the health system and people with relatively little need for healthcare.

The recurring themes of using publicly reported performance information for engaging in collective health decision making, selecting a provider, or advocating for one's own care align with roles identified in the literature on public involvement in healthcare decision making.^11^, ^41^, ^42^ Studies have identified that the public interface with the healthcare system in their role as a citizen, patient and consumer. For example, as a citizen, people are concerned about the financing of healthcare services and the services offered in their communities. The consumer is one in which people are expected to make choices of where or with whom to seek medical care before a therapeutic relationship is established.^20^, ^43^ As a patient, people evaluate whether services meet their individual or family members’ needs and make decisions for their own care usually in partnership with their healthcare provider.^11^, ^41^ However, the focus on primary care as opposed to other sectors of the health system as well as the local context and culture may have influenced the relative support for these roles in distinct ways.

### 4.1 The citizen role and public reporting

Our findings suggest that public performance reporting in primary care might promote the responsiveness of the health system through increased accountability and democratic participation, stimulating advocacy for high‐quality community healthcare services. Participants believed that governments had the responsibility to ensure equitable access to quality primary care and could be responsive to public lobbying. This type of citizen‐advocate engagement has been suggested as a mechanism for accountability in a publicly financed health system where most clinician‐level costs are not borne by the individual, thus limiting the influence of market forces.^23^

It is possible that the citizen‐advocate role was more dominant in this study because participants were volunteers who chose to engage in a full‐day session in their own region and were thus more likely than the general population to care about their community's healthcare services. There is little evidence on the public's actual or desired level of engagement in primary care advocacy for their community. However, a recent study surveying members of the public in Sweden and the UK found a stronger desire for participation in regional decision making for health services in Sweden (55%) compared to the UK (33%), suggesting that cultural and/or health system context may impact citizen health advocacy, including lower overall satisfaction with the health system in Sweden.^44^

Across Canada, the publicly funded health system is seen as a valued national social service^32^ and a civic entitlement. Individuals expect the public system to provide their needed care. However, Canadian seniors report being less satisfied than their peers in other countries on the overall performance of their health system.^45^ Canadians also believe citizens should demand more accountability from the health system and advocate more for better health services.^32^

Primary care performance reporting to the public which aims to promote citizen‐advocate engagement as a mechanism for accountability could optimize public interest and use of information by addressing people's expectation for community‐level civic engagement. Current trends in performance reporting recognize the importance of incorporating local context,^46^ and regional reporting approaches have been used in a number of countries such as in Australia's online primary care performance reports searchable by zipcode.^47^

Reporting performance information at the community level could facilitate civic engagement by empowering members of the public to understand how their community is served and to advocate for healthcare solutions for their own neighbourhoods. Further, linking performance information with local accountable decision‐makers, especially beyond government representatives, such as regional health administrators or primary care network leaders, might further support public engagement in provinces where decentralized decision making is being promoted.

### 4.2 The consumer role and public reporting

Participants perceived lack of choice of primary care provider was a major barrier to people anticipating using comparative primary care performance information about providers in a consumer role to choose the best provider for their needs. This finding across the three regions was influenced by the shared perception that people had a limited choice of primary care providers despite almost all participants having their own primary care physician and significant differences in rates of family physicians per 100 000 population. This may explain why participants did not strongly support the notion that primary care performance information should be used to stimulate competition among providers. This finding contrasts with much of the efforts to promote public performance information in many countries including the UK^48^ and the USA^49^ which seek to empower consumers to choose the best primary care provider for themselves or their family.

Similarly, the perception of limited primary care resources may have generated concern about the potential unintended consequences of public reports stimulating “doctor shopping,” and the potential for exacerbating inequities in access in regions.

Our findings also revealed an apparent tension between patient and consumer perspectives in using information on the performance of primary care. A consumer is expected to make informed choices in selecting care based on data from many patients’ experiences. However, individuals may discount objective data in the context of an existing relationship with a clinician, having invested time developing a connection with a clinician or place of care. These findings also highlight the unique features of primary care in terms of longitudinal relationships and care for a wide range of needs in partnership with patients.^42^ A relationship with one's own clinician raises the cost of changing providers and, therefore, diminishes the value of choice for those who are content with their care.

Further, the finding that many participants did not expect to engage with the performance data themselves but expected that it would be valuable to have it publicly reported to enable provider‐driven improvement of noted weaknesses, or third party oversight to ensure quality, suggests that performance reporting must actively seek to engage members of the public. Attempting to empower patients to vote with their feet may be too simplistic, and a focus on greater public awareness of variations in performance and the significance of such variations may more effectively lead to greater demand for high‐quality care as patients understand how public reports might complement their experience and knowledge of a provider which often rests heavily on their relationship with their primary provider.^12^, ^50^ Additionally, current reports on primary care performance could acknowledge that some people may not have a choice of provider and explain other ways people may use information to enhance their healthcare.^51^

### 4.3 The patient experience and public reporting

Our findings also suggest that public reporting may contribute to better patient experience by promoting awareness of optimal care which people should expect, as well as by enhancing trust in clinicians and the overall health system. While continuing professional development activities have not been a major target of performance reporting, our findings suggest that this information may be empowering to patients, warranting further exploration. The finding that some participants also felt that performance information should be publicly reported to promote quality assurance suggests that some people might value public performance reporting to empower their trust rather than their decision making. This rationale for public reporting, however, relies on the data being used effectively by providers, professional organizations or quality assurance groups. This suggests that primary care performance measurement and reporting efforts should focus on building capacity to use such data for quality improvement as well as for accountability. This is a key component of “intelligent transparency” driving renewal for public reporting in the UK.^52^ It would also mean that providers should be considered a target audience for public reporting alongside their patients and the public.

## 5 CONCLUSION

Public reporting on primary care performance to promote accountability, democratic participation and quality is receiving increasing attention as evidence continues to grow on its importance to population health, health system efficiency and individuals' experience within the healthcare system. Our findings suggest that members of the public may value and use public performance reporting to assess equity across the system in how their region is served and to advocate for their own community. Encouraging the public to utilize primary care performance reports to select optimal providers for themselves or ensure best care with their providers may require education not just on how to understand the data but also on the significance of the measures. Increasing the public's engagement in primary care as citizens, consumers and patients demands investment in effective measurement and reporting systems that enable people to effectively engage with this information, how and when they are most likely to use it. In Canada, public performance reporting to empower the public to advocate for their own communities’ primary care may be a particularly meaningful accountability mechanism to promote responsive healthcare.

## CONFLICT OF INTEREST

None declared.

## Data Availability

The data that support the findings of this study are available from the corresponding author upon reasonable request.
