# Supplementary material for: Citizen perspectives on the use of publicly reported primary care performance information: Results from citizen‐patient dialogues in three Canadian provinces
Source: Health Expect. 2019 May 10;22(5):974–82. doi: 10.1111/hex.12902 (PMC6803417; doi:10.1111/hex.12902)
Supplement: Supplementary file 1 [file HEX-22-974-s001.docx]

APPENDIX S1

Participant activity to assess the importance of collecting and reporting on primary care performance dimensions and select indicators

Participants were provided with the following written questions and definitions for each term in a glossary and were asked to discuss their answers with the group. For each question, they were asked to provide a ranking to assess the importance (not at all important, slightly important, important, fairly important, very important, no opinion) for each listed dimension.

*How important do you think it is to collect and report information about the following areas so that you can judge how good the care is in your community, or in your doctor or nurse practitioner’s office, or to help you get the best care for yourself or a family member?*

- *Access*
- *Patient-centred care*
- *Continuity*
- *Comprehensiveness*
- *Technical quality of care*
- *Safety*
- *Service integration*
- *Health equity*
- *Other areas of care that are important to collect and report on…*

They were then asked to assess the importance (same response categories as above) of collecting and reporting on specific indicators for select performance dimensions (access, patient-centred care, technical quality of care).

*How important do you think it is to collect and report on the following information about access to care so that you can judge how good the care is in your community or in your doctor or nurse practitioner’s office or to help you get the best care for yourself or a family member?*

- *Patients have a regular family physician or nurse practitioner that they see for check-ups or when they are sick.*
- *Patients can see their family physician or nurse practitioner on the same or next day when they call for an appointment.*
- *Patient can get medical care in the evening, on a weekend or on a public holiday through their family physician or nurse practitioner.*
- *Patients can call their regular family physician’s office with a medical question or concern during regular office hours, and get an answer on the same day.*
- *Other information about access to care that is important to collect and report on…*

*How important do you think it is to collect and report on the following information about patient-centred care so that you can judge how good the care is in your community or in your doctor or nurse practitioner’s office or to help you get the best care for yourself or a family member?*

- *Patients think their family physician or nurse practitioner always explains things in a way that is easy to understand*
- *Patients think that their family physician or nurse practitioner spends enough time with them.*
- *Patients think their family physician or nurse practitioner involves them as much as they want in decisions about their care or treatment.*
- *After seeing their family physician or nurse practitioner, patients feel more confident in dealing with their health or problems than before their visit.*
- *Other information about person-centred care that is important to collect and report on…*

*How important do you think it is to collect and report on the following information about the technical quality of care so that you can judge how good the care is in your community or in your doctor or nurse practitioner’s office or to help you get the best care for yourself or a family member?*

- *Patients are offered screening for various health problems according to recommended practice.*
- *Family physicians, nurse practitioners or an appropriate person in the office discusses the impact of healthy and non-healthy foods on patients’ health.*
- *Patients with diabetes have their blood sugar at the recommended target level.*
- *Patient with high blood pressure had a blood pressure check recorded in the last year.*
- *Other information about technical quality of care that is important to collect and report on…*
